# “Bioinformatics: Introduction and Methods,” a Bilingual Massive Open Online Course (MOOC) as a New Example for Global Bioinformatics Education

**DOI:** 10.1371/journal.pcbi.1003955

**Published:** 2014-12-11

**Authors:** Yang Ding, Meng Wang, Yao He, Adam Yongxin Ye, Xiaoxu Yang, Fenglin Liu, Yuqi Meng, Ge Gao, Liping Wei

**Affiliations:** 1 Center for Bioinformatics, State Key Laboratory of Protein and Plant Gene Research, School of Life Sciences, Peking University, Beijing, People's Republic of China; 2 National Institute of Biological Sciences, Beijing, People's Republic of China; 3 Peking-Tsinghua Center for Life Sciences, Academy for Advanced Interdisciplinary Studies, Peking University, Beijing, People's Republic of China; University of British Columbia, Canada

## Abstract

Bioinformatics is a fast-growing interdisciplinary field in which the demand for quality education exceeds the supply, especially in developing regions and countries. A massive open online course (MOOC) is a new model for education that delivers videotaped lectures and other course materials over the Internet for all interested persons around the globe to learn for free. Here we present our MOOC “Bioinformatics: Introduction and Methods,” which is the second bioinformatics MOOC in the world and one of the first batch of seven MOOCs from China. In the first two runs of this bilingual MOOC, more than 30,000 students with diverse backgrounds registered from 110 countries and regions. In this manuscript, we present the content design of the MOOC, the demographic profiles and learning patterns of the students, the requirement for English support, and feedback from on-campus students. We offer a few suggestions to other scientists who may be interested in creating a MOOC. We also remember the S* course, a successful open online bioinformatics course that ran from 2001 to 2007, long before the current wave of MOOCs. We believe that MOOC education has great potential to enhance global bioinformatics education.

## Introduction

Bioinformatics is a rapidly growing interdisciplinary field driven by extraordinary development in both life sciences and computer sciences in the past four decades. Exponentially growing amounts of biological data are generated by high-throughput biological technologies, especially next-generation sequencing. The total number of nucleotides sequenced now exceeds 2*10^15^ and doubles every five months. These “big data” have brought unprecedented opportunities for new discoveries, new patterns, and new hypotheses. At the same time, the data are heterogeneous and noisy. Handling big data requires advanced, efficient, and accurate computational methods and computer tools. Bioinformatics is a powerful technology for the efficient management, search, and analysis of petabytes of biological data. It is also a top-down, genome-scale, system-oriented methodology that complements traditional experimental methodologies in life sciences research.

In the early 1990s, only a handful of bioinformatics degree programs existed, mostly in the United States and the United Kingdom. Today there are hundreds around the world (see list at www.iscb.org). In mainland China, the first bioinformatics center was founded at Peking University in 1997, and today bioinformatics degree programs are available at 30 universities and institutes in 16 cities (S1 Table). Nevertheless, the demand for quality bioinformatics education far exceeds the supply. There is still a shortage of bioinformatics education programs in many universities, and this shortage is especially severe in developing countries and regions.

The Massive Open Online Course (MOOC) is a new model for education that delivers videotaped lectures and other course materials over the Internet for all interested persons around the globe to learn for free. The two largest platforms for MOOCs are Coursera (www.coursera.org) and edX (www.edx.org). Within a few short years, MOOCs have reached tens of millions of people from all over the world and have begun to transform higher education. MOOCs differ from previous generations of online education in several ways. MOOCs are created and officially endorsed by major universities and institutes and are usually based on on-campus courses from college-level or graduate-level curricula. Instructors and students interact in online discussion forums, assignments and exams are graded, and certificates of completion are issued to students who satisfy predefined performance criteria. The improvement of Internet technologies such as increased bandwidth and streaming media has made it possible to distribute high-definition videos across the web smoothly, which has significantly improved user experience.

In response to the need for advancing global bioinformatics education, we took advantage of the new MOOC technology platforms and created a new MOOC on Coursera entitled “Bioinformatics: Introduction and Methods” (available: https://www.coursera.org/course/pkubioinfo). It is the second MOOC in bioinformatics and computational biology in the world. The first run of this MOOC was in fall 2013, and the second run was in spring 2014. A total of 18,367 students from 105 countries and regions registered for the MOOC in the first run, and a total of 16,714 students from 63 countries and regions registered for the second run. To better understand the student population, we collected and analyzed student demographic, activity, and survey data. We hope that our experiences and lessons might be useful for others interested in MOOC education. Our MOOC is also unique in that it is among the first batch of seven MOOCs from mainland China and the only one that is fully bilingual—the lectures were taught in both English and Chinese with subtitles also in English and Chinese. The lessons we learned may offer an interesting international perspective on MOOC education.

## Content Design of the MOOC

We designed this MOOC, “Bioinformatics: Introduction and Methods,” to teach the fundamental concepts and computational methods in bioinformatics and their applications in life sciences. We intended it to be followed by another MOOC on advanced bioinformatics in the near future. In addition to the instructors (Gao and Wei), the teaching staff also consists of four teaching assistants (TAs) (Ding, Wang, He, and Ye) and three online discussion forum TAs (Yang, Liu, and Meng). This MOOC is primarily based on the “Methods in Bioinformatics” course that the instructors created in 2006 and taught in every fall semester from 2006 to 2013 at Peking University. It was designed for undergraduates and first-year graduate students in bioinformatics, molecular biology, genetics, computer science, mathematics, statistics, or physics. Students should have a basic knowledge of molecular biology and computational sciences, including familiarity with basic terms such as “gene,” “genome,” “expression,” and “regulation,” essential concepts such as probability theory and linear algebra, and one programming language.

The MOOC was divided into 12 topics, shown in Table 1. These topics were further divided into 43 units, each of which was covered by a lecture video. We started with basic sequence alignment, sequence database search, and sequence motif search, and moved on to next-generation sequencing analysis. We then described what we could do with the sequencing data, i.e., functional prediction of genetic variants and prediction and analysis of noncoding RNAs. We discussed the concept and implementation of ontology and the identification of molecular pathways. To provide a useful future reference for the students, we reviewed existing bioinformatics database and software resources. Finally, we used two case studies to demonstrate how to use bioinformatics data and analysis to study interesting biological questions.

**Table 1:** Syllabus of the MOOC “Bioinformatics: Introduction and Methods.”

Week	Topic
1	Introduction and history of bioinformatics
2	Sequence alignment
3	Sequence database search and genome-wide alignment
4	Sequence motif building and motif search
5	Next-generation sequencing (NGS): mapping of reads from resequencing and calling of genetic variants
6	Next-generation sequencing (NGS): transcriptome analysis and RNA-Seq
7	Functional prediction of genetic variants
8	Prediction and analysis of noncoding RNAs
9	Ontology and identification of molecular pathways
10	Bioinformatics database and software resources
11	Case study I: Origination and evolution of new genes
12	Case study II: Evolutionary functional analysis of DNA methyltransferase

In addition to the main lecture videos, we also provided 25 supplementary learning videos to cater to the different needs of the students. These include additional background knowledge such as the basics of databases. They also include a step-by-step demonstration of the bioinformatics software whose methods were taught in the main video, narrated videos of running next-generation sequencers, and interviews with pioneers such as Dr. Michael Waterman from the University of Southern California, who recalled how he and Dr. Temple Smith developed the Smith-Waterman algorithm; Dr. Maynard Olson from University of Washington, who recalled bioinformatics' contribution to the Human Genome Project; and Dr. Gang Pei from Tongji University, who discussed his view of bioinformatics' role in life sciences. Also included as supplementary learning materials are videos of seminal paper presentations by students taking our on-campus course. The supplementary materials were not tested in the quizzes or exams. The students of an interdisciplinary field such as bioinformatics tend to have diverse backgrounds. The MOOC platform provided flexibility to provide these materials to cater to different learning needs of students.

To date, we have run this MOOC twice on Coursera. The first run in the fall of 2013 lasted 12 weeks, but Coursera staff suggested shortening the course. According to their data, longer MOOCs tended to lose more students. Therefore, the second run in the spring of 2014 covered the same materials in six weeks, with two topics per week. For instance, materials covered in Week 1 and Week 2 in the first run were made into Week 1 Topic 1 and Week 1 Topic 2. Our analysis of student data did not find significant differences between the two runs (see below). The students were graded on quizzes and a final exam. Students who received a final grade higher than 70 (in the first run, 60 in the second run) received a Certificate of Completion signed by the instructors with authorization by Peking University. Students who received a final grade higher than 95 (in the first run, 85 in the second run) received a Certificate of Completion with Distinction.

## Demographic Profiles and Learning Patterns of the Students

The demographic profile and learning patterns of MOOC students are likely to differ from those of students on campus. To better understand the MOOC student population, we downloaded Coursera's backend SQL (Structured Query Language) data, which details the activity of every registered student. We also ran a number of student surveys focusing on different aspects of the course. We collected data from both runs of the MOOC and compared them when possible. The first run and the second run showed largely consistent patterns.

As shown in Table 2, a total of 18,367 students registered for the first run of the MOOC and 16,714 for the second run. Of them, 11,946 (65.04%) and 8,352 (49.97%) watched at least one main video. The distributions of the final grades were not normal, and the histograms of the nonzero grades showed two peaks near both ends (S1 Figure). In the end 520 (2.83%) students passed and received a certificate in the first run, including 142 who received a Certificate of Completion with Distinction; similarly, in the second run 510 (3.05%) passed and received a certificate, of whom 156 received Certificate of Completion with Distinction (Table 2). These percentages are comparable to those of other MOOCs available on Coursera so far (personal communication with Coursera staff. See Acknowledgments). In contrast, of the 91 students who registered in the Signature Track of our second run and paid US$49 for taking the MOOC (which was free for all other students), 64 (70.33%) received a Verified Certificate of Completion and 47 (51.65% of all these 91 students) received a Verified Certificate of Completion with Distinction. Interestingly and importantly, another 491 (2.67%) and 341 (2.04%) students in the first and second run, respectively, watched every main lecture video but did not take any of the quizzes or exam and thus received a final score of zero. Of this group, 287 (1.56%) and 257 (1.54%), respectively, watched not only all main lecture videos but also all supplementary videos. This group of over 800 learners was apparently interested in the content but not the certificate.

**Table 2:** Overall statistics of the students.

	Number (Percentage)	
	First run	Second run
**Students who registered**	18,367 (100%)	16,714 (100%)
**Students who watched at least one course video**	11,946 (65.04%)	8,352 (49.97%)
**Students who signed up for the Signature Track**	N/A*	91 (0.54%)
**Students who obtained a Certificate of Completion**	520 (2.83%)	510 (3.05%)
**Students who obtained a Certificate of Completion with Distinction**	142 (0.77%)	156 (0.93%)
**Students who signed up for the Signature Track and obtained a Certificate of Completion**	N/A*	64 (0.38% of all students; 70.33% of all students that signed up for Signature Track)
**Students who signed up for Signature Track and obtained Certificate of Completion with Distinction**	N/A*	47 (0.28% of all students; 51.65% of all students that signed up for Signature Track)
**Students who scored zero but watched all the main lecture videos**	491 (2.67%)	341 (2.04%)
**Students who scored zero but watched all the main and supplementary videos**	287 (1.56%)	257 (1.54%)

* N/A, not applicable.

**Figure 1:**
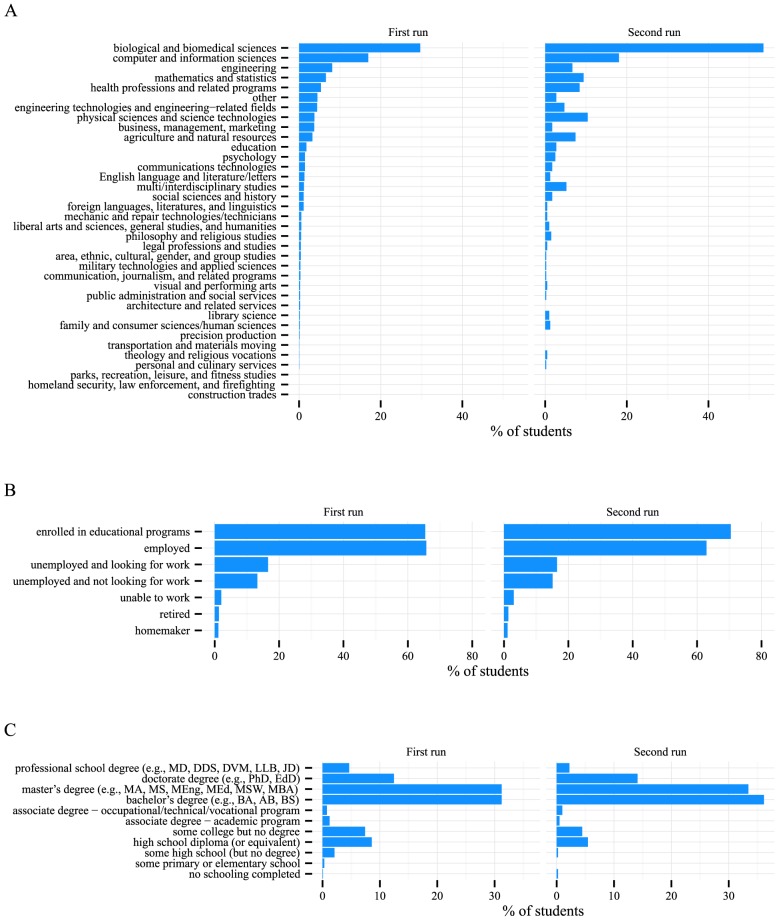
Students of the MOOC had diverse background. (A) Students came from a broad range of disciplines. (B) More than half of the students were currently enrolled in educational programs or were currently working. (C) Most students had acquired a Bachelor's or Master's degree.

We analyzed the background of the MOOC students. Bioinformatics is an interdisciplinary field, and we as instructors are used to having students with diverse backgrounds. However, the backgrounds of the students of MOOCs are far more diverse than what we are familiar with. As shown in Fig. 1A, the students came from a broad range of academic backgrounds. The most prevalent background was “biological and biomedical sciences” (29.67% and 53.47% in the first and second run, respectively), followed by “computer and information sciences” (16.95% and 18.07%), “engineering” (8.11% and 6.68%), “mathematics and statistics” (6.57% and 9.41%), and “health professionals and related programs” (5.34% and 8.42%). This pattern reflects the interdisciplinary nature of bioinformatics. Fig. 1B shows that 65.39% and 70.47% of the students in the first and second run, respectively, were enrolled in educational programs, and 65.72% and 62.91% of students were employed. Fig. 1C shows that the students' highest degree was typically a Bachelor's or Master's, followed by PhDs and a broad range of other educational levels.

**Figure 2:**
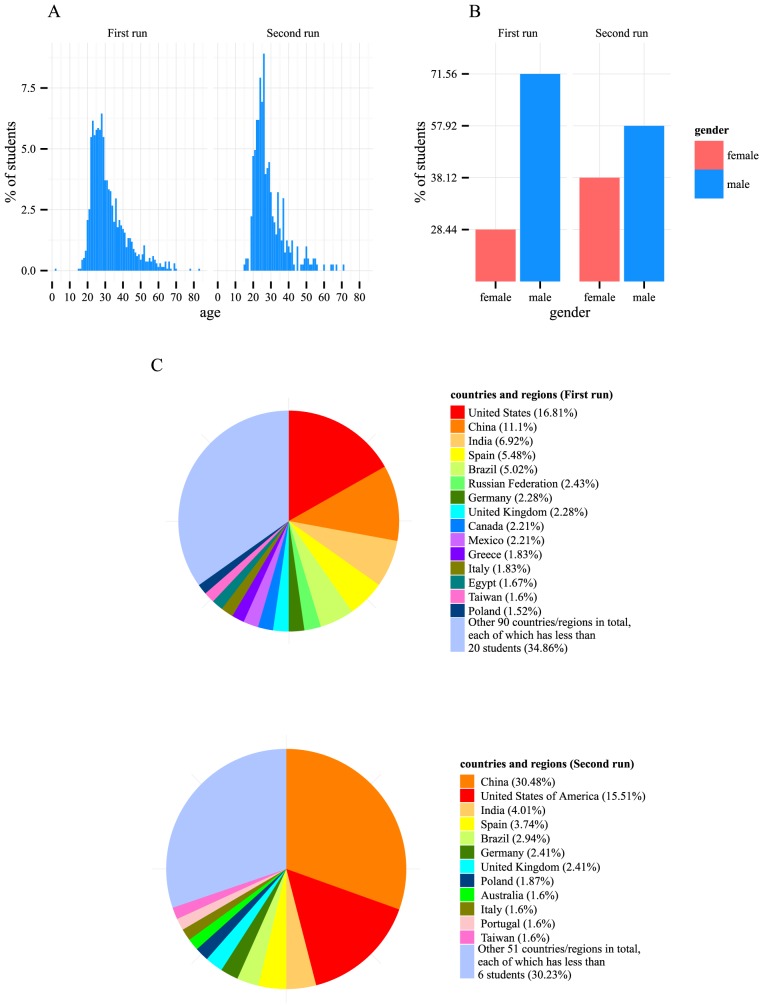
Students' demographic profiles were diverse. (A) The most prevalent age group consisted of students in their twenties. (B) There were more male students than female students. (C) Students came from over 100 countries and regions.

Analysis of the demographic data of the students showed that the age distribution was skewed toward people in their twenties (Fig. 2A). There were more male students than female students, with a male-to-female ratio of 2.52 and 1.52 in the first and second runs, respectively (Fig. 2B). The student body was highly international and included students from 105 countries and regions in the first run and 63 countries and regions in the second run. The most highly represented countries and regions were China (11.10% and 30.48% in the first and second runs, respectively) and the US (16.81% and 15.51%), followed by India, Spain, Brazil, Germany, the UK, the Russian Federation, and Poland (Fig. 2C).

## English Dubbing and Subtitles in a Bilingual MOOC

**Figure 3:**
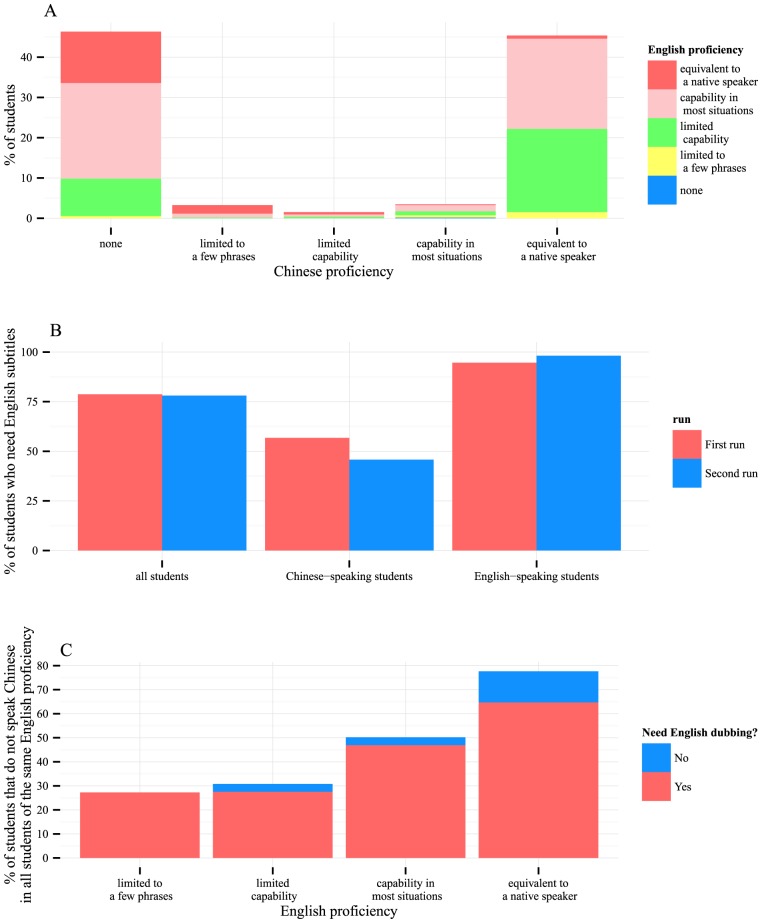
English support is needed in an international MOOC. (A) About half of the students did not know Chinese. (B) English subtitles were welcomed by both English- and Chinese-speaking students. (C) With English subtitles available, over 80% of non-Chinese-speaking students requested English dubbing.

In May 2013, Peking University and Tsinghua University became the first two universities in mainland China to announce that they would begin creating MOOCs. Our MOOC is one of the first batch of seven MOOCs created in mainland China, all from Peking University. As shown in Fig. 3A, about a quarter of the students speak mostly Chinese with limited English, thus MOOCs taught in their native language remove the language barrier and allow them to focus on learning the content better. In addition, our MOOC was the first MOOC ever taken by many of the Chinese students (personal communication with Coursera staff. See Acknowledgments). Thus, MOOCs taught in languages other than English can help promote MOOCs to a broader worldwide audience. At the same time, as we showed in Fig. 3A, about half of the students did not know any Chinese. Thus, providing English translations was necessary. This can be done with subtitles and/or dubbing. The first half of the first run of our MOOC was taught in Chinese with slides in English and subtitles in both Chinese and English. Our MOOC would not have been useful for the large population of students who did not know Chinese if we had not provided English subtitles. Interestingly, our surveys indicated that English subtitles were considered necessary by not only English-speaking students but also many Chinese-speaking students (Fig. 3B). A survey run during the first half of the first run of the MOOC showed that, when the English subtitles were available, a total of 89.96% of the students who did not speak Chinese preferred to also have English dubbing and 10.04% were satisfied with just English subtitles. The preference for English dubbing seemed stronger among students who were not native English speakers (Fig. 3C). Thus, we added English dubbing in the second run of the MOOC, making it bilingual.

**Figure 4:**
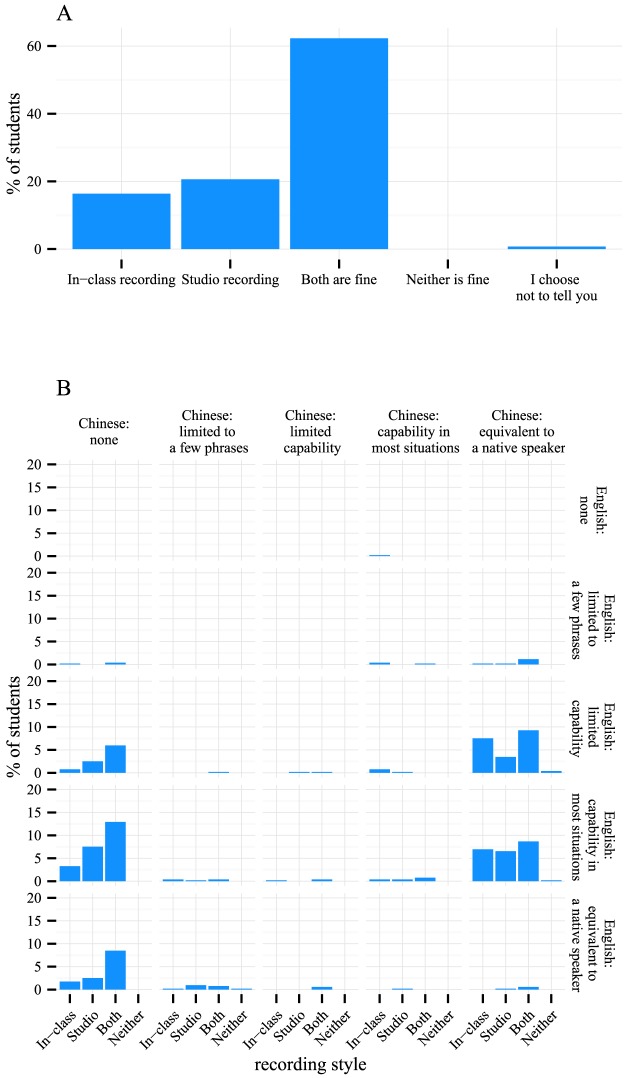
Students' preference for in-class recording versus studio recording. (A) Half of the students were fine with both styles. The other half had different preferences. (B) Subtle but meaningful differences in preference of recording styles exist between English- and Chinese-speaking students.

We also experimented with two videotaping styles at the beginning of the first run: in-class recording and studio recording. The lectures were taught in Chinese with subtitles in both Chinese and English at the time of the survey. In-class recording meant that the video was captured when the instructor was teaching the lecture live on campus. Studio recording was done when the instructor recorded his or her voice and captured the computer screen simultaneously in a studio or office, without capturing the instructors' faces. The main differences between the two styles are that in the in-class recording style the instructor was shown from time to time and his or her voice seemed more colloquial and natural, whereas in the studio recording style, only screen captures were shown and the instructor's words were more accurate but monotonic. We surveyed the preference of the students. Half of the students were fine with both styles, and about equal numbers of students had a strong preference for one style or the other (Fig. 4A). When we correlated preference with language proficiency, we found that students who speak Chinese but not English had a stronger preference for in-class recording, whereas students who speak English but not Chinese had a stronger preference for studio recording (Fig. 4B). Since the lectures were taught in Chinese, we speculate that the Chinese-speaking students might have preferred the more natural voices and images in the in-class recording, whereas the images of someone speaking a foreign language might have been a distraction for the English-speaking students who did not understand Chinese.

## Using the Survey Function to Learn about the Students' Opinions

**Figure 5:**
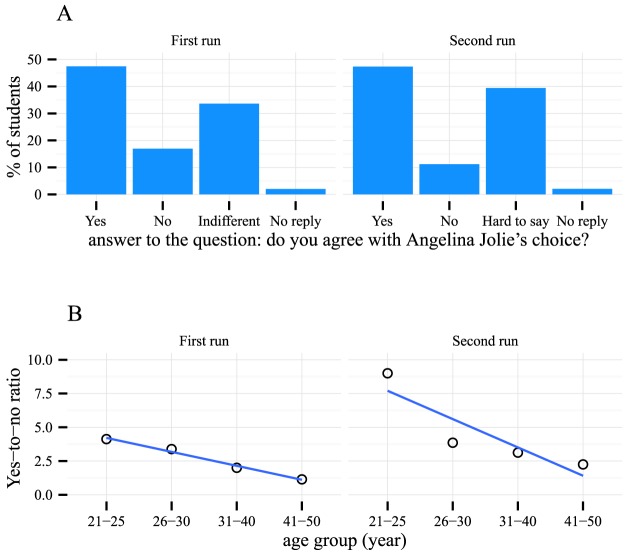
Students' opinions of Angelina Jolie's choice to do a mastectomy. (A) About half of the students supported Jolie's choice in both runs. (B) Older students seemed more conservative than younger students about this choice.

We have found the survey function of the Coursera MOOC platform to be useful for learning about the background and opinions of the students. For instance, as part of the topic “Functional prediction of genetic variants,” in order to encourage the students to think about the real-life implications of the knowledge they had gained, we ran a survey to poll the students' opinions about a highly publicized medical decision made by the actress Angelina Jolie, who has a strong family history of cancer and chose to undergo a mastectomy after being found to carry a mutation in *BRCA1*. About half of the students agreed with Jolie's choice, and 11%–16% disagreed (Fig. 5A). There was no difference between the answers given by male and female students. There was, however, an apparent negative correlation between the students' age group and the percentage that agreed with Jolie's choice, as shown in Fig. 5B (first run: linear regression *p*-value = 0.00668; second run: linear regression *p*-value = 0.107).

## Discussion

MOOCs are a highly effective channel for global education. In the first two runs of our MOOC, over 30,000 students from over 100 countries and regions registered, of whom 1,030 students received a Certificate of Completion. Another 831 students watched every video. In comparison, the on-campus course “Methods in Bioinformatics” taught by the instructors (Gao and Wei) had a total of 800 registered students from 2006 until the present, about 90%–95% of whom passed. The MOOC has reached far more students within a short time. It is especially valuable for students in universities in which bioinformatics courses are not available. One of the students from the Russian Federation in the first run of our MOOC stated the following: “Thank you for this course. In my city there [are] no universities that provide bioinformatics courses and no professors who can teach bioinformatics, so that is the only chance for me [except for] reading books to get basic bioinformatic knowledge.”

In the fall of 2013, the instructors continued to teach the “Methods in Bioinformatics” course on the Peking University campus as usual, except that we used our MOOC as supplementary learning material. The on-campus lectures covered the same materials as those covered by the main lecture videos of the MOOC. However, the on-campus students were required to give group presentations on seminal papers as part of their final grade, in addition to the assignments and exams. A survey of the on-campus students showed that 88.46% students agreed that they could learn the course more effectively when the MOOC was available, and 84.62% students agreed that online learning complemented face-to-face learning. When asked about specific functions of the MOOC that they found most useful, 53.85% chose the online discussion forum, and 78.85% chose the supplementary materials. Next fall we plan to experiment with the “flipped classroom” approach in which we will ask the on-campus students to watch the MOOC first each week; we will then use the class time for guided discussions and student presentations.

We will create a MOOC on advanced bioinformatics in the near future, and we also encourage interested students to register for other MOOCs. As of July 28, 2014, according to www.mooc-list.com, 167 MOOCs were available in all areas of biology. We identified 14 available and upcoming MOOCs on bioinformatics, computational biology, systems biology, computational evolution, and computational neuroscience, shown in Table 3, including the first bioinformatics MOOC, “Bioinformatics Algorithms (Part 1),” from the University of California, San Diego. We also refer interested readers to a recent article by David Searls that provided a comprehensive list of online resources including not only MOOCs but also other online materials related to computational biology [1]. Some topics are not yet covered in sufficient depth by the available and upcoming MOOCs, such as structural bioinformatics. New MOOCs on these topics would be welcome additions to the global bioinformatics education.

**Table 3:** Existing and upcoming MOOCs on bioinformatics and computational biology.

Course name	Platform	University	Start Date and Length of the Course	Instructors
Bioinformatics Algorithms (Part 1)	Coursera	University of California, San Diego	First run: November 4, 2013; 14 weeks. Second run: September 15, 2014; 12 weeks	Phillip E. C. Compeau, Nikolay Vyahhi, Pavel Pevzner
Bioinformatics Algorithms (Part 2)	Coursera	University of California, San Diego	January 12, 2015; 12 weeks	Pavel Pevzner, Phillip E. C. Compeau
Bioinformatics: Introduction and Methods	Coursera	Peking University	First run: September 30, 2013; 12 weeks. Second run: March 17, 2014; 6 weeks	Ge Gao, Liping Wei
Bioinformatic Methods I	Coursera	University of Toronto	January 6, 2014; 6 weeks	Nicholas James Provart
Bioinformatic Methods II	Coursera	University of Toronto	March 3, 2014; 6 weeks	Nicholas James Provart
Introduction to Systems Biology	Coursera	Icahn School of Medicine at Mount Sinai	First run: March 31, 2014; 10 weeks. Second run: September 2, 2014; 9 weeks and 3 days	Ravi Iyengar
Dynamical Modeling Methods for Systems Biology	Coursera	Icahn School of Medicine at Mount Sinai	Second run: March 2, 2015; 7 weeks	Eric Sobie
Experimental Methods in Systems Biology*	Coursera	Icahn School of Medicine at Mount Sinai	October 27, 2014; 7 weeks	Marc Birtwistle
Network Analysis in Systems Biology	Coursera	Icahn School of Medicine at Mount Sinai	January 5, 2015; 7 weeks	Avi Ma'ayan
Integrated Analysis in Systems Biology*	Coursera	Icahn School of Medicine at Mount Sinai	April 20, 2015; 4 weeks	Susana Neves, Ravi Iyengar
Data Analysis for Genomics	edX	Harvard University	April 7, 2014; 9 weeks	Rafael Irizarry, Michael Love
Bioinformatics: Life Sciences on Your Computer	Coursera	Johns Hopkins University	June 9, 2014; 5 weeks	Bob Lessick
Computational Molecular Evolution	Coursera	Technical University of Denmark	Jan 13, 2014; 6 weeks	Anders Gorm Pedersen
Computational Neuroscience	Coursera	University of Washington	To be determined**; 8 weeks	Rajesh P. N. Rao, Adrienne Fairhall

* Not available on www.mooc-list.com before July 28, 2014.

** This course had no available session at the time we checked its Coursera homepage (September 13, 2014).

Creating the MOOC was a rewarding yet challenging experience for the teaching team. We have a few suggestions for other instructors who may be interested in converting their on-campus courses to MOOCs. Although each on-campus lecture is typically ∼1–2 hours long, online students often need to fit the MOOCs into their busy schedule and they typically do not have that big of a chunk of time. Thus, a topic in a MOOC needs to be broken up into short units of ∼10 minutes each. Compared to on-campus students, students of the MOOCs have even more diverse backgrounds, uneven education levels, and may speak different languages. Not being able to see the students' faces can make it difficult to assess and respond to their learning needs in real time. Fortunately, the MOOC platform has its own advantages. Supplementary learning videos are a great resource to cater to the different needs of the students. The survey function and the online discussion forum provide additional channels for instructors to learn about the students' backgrounds, needs, and opinions. Once a MOOC is created in one language, it can be translated into other languages and thereby reach a broader global audience. Sticking to the schedule of the MOOC is more important than trying to make it perfect, which may cause unnecessary delay. We assigned one TA or forum TA a day to monitor the discussion forum to avoid leaving students' important questions unanswered. It is essential to have dedicated and capable TAs with good time-management skills. Ideally, the TAs should be the instructors' own graduate students, as being a TA for the MOOC will inevitably interfere with his or her research for the duration of the MOOC. The workload involved in the first run of a MOOC can be quite high but is significantly lower in the second and future runs.

Last but not least, it is worth remembering that, from 2001 to 2007, long before the current wave of MOOCs, there was a successful open online bioinformatics course by S* Alliance (http://www.apbionet.org/s-star/index.html) [2]. S* was an alliance of eight different universities in five continents (S* was named because the original six member universities all had an “S” in their names). Fifteen lecturers from the member universities, including Nobel Laureate Michael Levitt from Stanford University and one of the authors of this manuscript, Liping Wei, then at Stanford University, recorded their lectures on a variety of topics in bioinformatics. The lectures, together with online assignments and online discussion forums, were made into a full online course that was run once or twice a year. Students who passed the course received a certificate. Hundreds to thousands of students from dozens of countries registered for each run of the S* course. These numbers were considered phenomenal in the early 2000s. The S* courses were delivered via the Integrated Virtual Learning Environment (IVLE) developed at the National University of Singapore. Slow internet connections were the main complaints from the students, so mirror sites were set up around the globe. Unfortunately, despite its success and innovation, the S* course stopped being offered after 2007 because of a funding shortage. In spirit and in practice, the S* course was a MOOC long before MOOCs existed.

In conclusion, we believe that, for a fast-growing discipline such as bioinformatics, MOOCs have great potential to advance global education. We expect to see more and more MOOCs in bioinformatics and related fields. In the near future, the full curriculum of bioinformatics may become available through an ever-expanding collection of MOOCs, benefiting all interested learners around the globe.

## Supporting Information

S1 Figure Histograms of nonzero final grades for the first and second run. Each bin excludes its lower bound and includes its upper bound; for example, the bin (10, 20) contains all students with a grade more than 10 and no more than 20.
(EPS)

S1 Table Bioinformatics degree programs in mainland China.
(DOCX)
