# Supplementary material for: “Bioinformatics: Introduction and Methods,” a Bilingual Massive Open Online Course (MOOC) as a New Example for Global Bioinformatics Education
Source: PLoS Comput Biol. 2014 Dec 11;10(12):e1003955. doi: 10.1371/journal.pcbi.1003955 (PMC4263353; doi:10.1371/journal.pcbi.1003955)
Supplement: S1 Table — Bioinformatics degree programs in mainland China. (DOCX) [file pcbi.1003955.s002.docx]

**Table S1.** Bioinformatics degree programs in mainland China

| **City** | **University/Institute** | **School/Center** | **Degrees offered** |
| --- | --- | --- | --- |
| Beijing | Peking University | Center for Bioinformatics, School of Life Sciences | Ph.D. |
|  |  | Center for Quantitative Biology | Ph.D. |
| Beijing | Tsinghua University | Department of Biological Sciences and Biotechnology | Ph.D. |
|  |  | Institute of Bioinformatics, Department of Automation | Ph.D. |
| Beijing | Chinese Academy of Sciences | Beijing Institute of Genomics | Ph.D. |
|  |  | Center of Systems Biology, Institute of Biophysics | Ph.D. |
|  |  | Center of Molecular Systems Biology, Institute of Genetics and Developmental Biology | Ph.D. |
| Beijing | China Agricultural University | College of Biological Sciences | Ph.D., Master |
| Beijing | Beijing Normal University | College of Life Sciences, Laboratory of Computational Molecular Biology | Master |
| Chengdu | Sichuan University | School of Life Sciences | Ph.D., Master |
| Chongqing | Chongqing University of Posts and Telecommunications | College of Bioinformation | Bachelor |
| Guangzhou | Sun Yat-sen University | Center for Bioinformatics, College of Life Sciences | Ph.D., Master |
| Hangzhou | Zhejiang University | School of Life Science, Institute of Bioinformatics | Bachelor |
| Harbin | Harbin Medical University | College of Bioinformatics Science and Technology | Master, Bachelor |
| Hefei | University of Science and Technology of China | School of Life Sciences | Ph.D., Master |
| Kunming | Yunnan University | School of Life Sciences | Master |
| Lanzhou | Lanzhou University | School of Life Sciences | Master |
| Nanjing | Nanjing University | School of Life Sciences | Ph.D., Master |
| Nanjing | Nanjing Agricultural University | Center for Bioinformatics, College of Life Sciences | Master |
| Nanjing | Southeast University | State Key Laboratory of Bioelectronics, School of Biological Science & Medical Engineering | Ph.D., Master |
| Nanjing | China Pharmaceutical University | School of Life Science and Technology | Ph.D., Master |
| Shanghai | Fudan University | School of Life Sciences | Ph.D., Master |
| Shanghai | Shanghai Institute for Biological Sciences | Key Laboratory of Systems Biology | Ph.D. |
| Shanghai | Tongji University | School of Life Science | Ph.D., Master, B.S. |
| Shanghai | Shanghai Jiao Tong University | Department of Biomedical Engineering, College of Life Science and Biotechnology | Master |
| Shanghai | East China Normal University | School of Life Sciences | Ph.D., Master |
| Shanghai | Shanghai Jiaotong University | School of Life Sciences | Master |
| Tianjin | Nankai University | College of Life Sciences | Ph.D., Master |
| Tianjin | Tianjin University | Tianjin University Bioinformatics Centre | Master |
| Wuhan | Huazhong Agricultural University | School of Life Sciences | Master, Bachelor |
| Wuhan | Huazhong University of Science and Technology | School of Life Science and Technology | Ph.D., Master, B.S. |
| Xiamen | Xiamen University | Department of Chemistry | Ph.D., Master |
| Xi’an | Xi’an Jiaotong University | School of Life Science, Institute of Bioinformatics | Ph.D., Master |
| Yangling | Northwest Agriculture and Forestry University | Center for Bioinformatics, College of Life Sciences | Ph.D., Master |
